# Confined Room-Temperature Ferromagnetism in Kagome (Fe_3_Sn_2_/CoSn) Superlattices

**DOI:** 10.1021/acsnano.6c03980

**Published:** 2026-08-06

**Authors:** Rajesh Dutta, Christopher J. Jensen, Tarush Tandon, Prajwal M. Laxmeesha, Alexander Velič, Murat Tuna Pamuk, Chuqiao Shi, Yu-Tsun Shao, Timothy R. Charlton, Jochen Stahn, Alexander J. Grutter, Julie A. Borchers, Steven J. May

**Affiliations:** † Department of Materials Science and Engineering, College of Engineering, 6527 Drexel University, Philadelphia, Pennsylvania 19104, United States; ‡ NIST Center for Neutron Research, 10833 National Institute of Standards and Technology, Gaithersburg, Maryland 20899-6102, United States; § Mork Family Department of Chemical Engineering and Materials Science, 5116 University of Southern California, Los Angeles, California 90089, United States; ∥ Neutron Sciences Division, 6146 Oak Ridge National Laboratory, Oak Ridge, Tennessee 37830, United States; ⊥ 419061 PSI Center for Neutron and Muon Sciences, Villigen PSI 5232, Switzerland

**Keywords:** kagome metals, topological materials, magnetism, superlattice, neutron reflectometry

## Abstract

We reveal the presence of robust ferromagnetism in topological kagome (Fe_3_Sn_2_/CoSn)_×*n*_ superlattices via polarized neutron reflectivity (PNR) and magnetization measurements. Using molecular beam epitaxy, we have synthesized superlattices of alternating ferromagnetic Fe_3_Sn_2_ and paramagnetic CoSn layers, the interfacial integrity of which is confirmed using scanning transmission electron microscopy. Magnetization depth profiles, obtained by fitting the PNR data, confirm the presence of ferromagnetism in the confined Fe_3_Sn_2_ layers at room temperature, with minimal thickness dependence down to 2 nm. A net magnetization is found in the near-surface region of CoSn, which we attribute to surface oxidation. Our results demonstrate that superlattice engineering is a means to confine ferromagnetism in kagome metals, enabling future studies of thickness-dependent anomalous Hall and Nernst effects as well as spin-dependent transport and tunneling in device structures.

## Graphical abstract

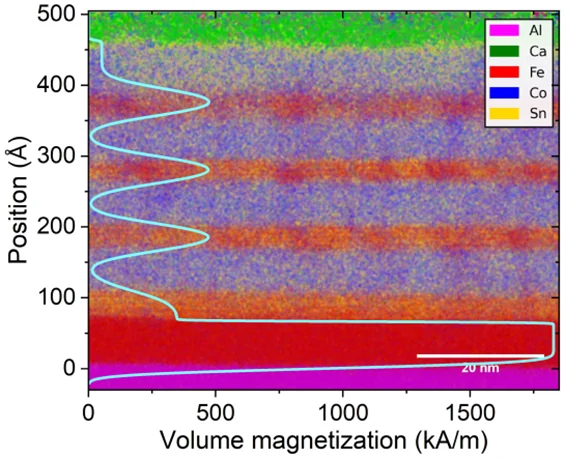

## Introduction

Kagome metals have emerged as an important family of quantum materials owing to their rich array of collectively ordered states and the presence of topological band features.^1^ These materials host planes of transition-metal atoms (*M*) arranged on a sublattice forming the geometric kagome pattern, often with group 13 to 15 elements (*X*) located in or near the central hexagonal site within the kagome layer. Examples of kagome metals include Fe_3_Sn_2_, FeSn, *R*Mn_6_Sn_6_ (*R* = rare earth), and *A*V_3_Sb_5_ (*A* = alkali metal), all of which host *M*_3_ *X* kagome planes.^2–6^ Recent works have demonstrated that kagome metals can exhibit an array of cooperative phenomena such as superconductivity, charge density waves, ordered magnetic states, and produce large spin, topological, and anomalous Hall effects.^7–14^ The stacking sequence of the kagome layers within these materials can have a profound influence on their macroscopic properties, as illustrated by the Fe–Sn binary kagome family.^2,15^ The compound Fe_3_Sn consists of kagome layers stacked consecutively along the *c*-axis. In Fe_3_Sn_2_, the Fe_3_Sn kagome layers form adjacent bilayers separated by a Sn_2_ monolayer (Fe_3_Sn/Fe_3_Sn/Sn_2_...). FeSn crystallizes with alternating Fe_3_Sn_2_ and Sn_2_ monolayers (Fe_3_Sn/Sn_2_/Fe_3_Sn/Sn_2_...). The presence of ferromagnetism within Fe_3_Sn and Fe_3_Sn_2_ correlates with adjacent stacking of the nearest-neighbor kagome planes.^16,17^ In contrast, FeSn is antiferromagnetic with ferromagnetic coupling within each kagome layer, but with antiparallel alignment of moments between adjacent kagome planes.^3^ Electronically, the band structure of FeSn bears a strong resemblance to the idealized electron dispersion obtained from a monolayer kagome model, with flat bands present above and below *E*_F_ and a Dirac cone at the K point.^2,3,18,19^ As the kagome layers become less electrically isolated in Fe_3_Sn_2_ and Fe_3_Sn, the band structure becomes increasingly complex with a higher density of bands while still retaining the signature flat bands and topological points that have generated such interest in kagome materials.^20–22^

The strong dependence of magnetism on how the Fe_3_Sn kagome layers are stacked motivates the use of epitaxial heterostructures, in which layer stacking can be controlled in the formation of superlattices as a promising direction to realize new physical states. To date, all members of the kagome Fe–Sn family have been deposited as monolithic thin films, yielding insights into the surface electronic structures, magnetic anisotropy, spin reorientation transitions, and higher-order Hall effects.^9,23–30^ In contrast, the growth and properties of kagome-based superlattices and complex heterostructures remain largely unexplored territory, despite the central role that interfaces play in the emergence of novel phenomena in quantum materials. We are aware of only one report of kagome superlattices in which dissimilar kagome materials are stacked along the growth direction. Cheng and coauthors used molecular beam epitaxy to grow (Fe_3_Sn_2_)/(Fe_3_Sn), demonstrating that discrete layers of these two materials could be realized with individual layer thicknesses as low as 2 nm.^31^ Expanding the families of epitaxial kagome superlattices that can be synthesized would enable new strategies for tuning electronic and magnetic behavior, such as confinement and finite thickness effects, magnetic exchange coupling, and interfacial electronic reconstructions.

Here, we demonstrate the growth of superlattices consisting of ferromagnetic Fe_3_Sn_2_ and paramagnetic CoSn, a kagome metal isostructural to FeSn, by using molecular beam epitaxy (MBE). The superlattices exhibit well-defined interfaces, as measured by scanning transmission electron microscopy (STEM) with energy-dispersive X-ray spectroscopy (EDS), X-ray diffraction, and polarized neutron reflectometry (PNR). Using polarized neutron reflectometry, we revealed a magnetization modulation along the growth direction between the Fe_3_Sn_2_ and CoSn layers. The saturation magnetization within the confined Fe_3_Sn_2_ layers is only slightly reduced from bulk values and is relatively insensitive to the thickness range probed in this study (between 2 and 8 nm). The magnetic depth profile largely follows the chemical depth profile, indicating that Fe_3_Sn_2_ retains ferromagnetic order up to the interface with CoSn and does not exhibit significant interfacial magnetic dead layers. This new superlattice system can serve as a platform for probing magnetism in the ultrathin (single-kagome-layer) limit and for understanding the potential implications of local charge transfer between kagome metals with different band fillings.

## Results and Discussion

We used MBE to grow (Fe_3_Sn_2_/CoSn) kagome superlattices on Fe-buffered Al_2_O_3_(0001) substrates. A schematic showing the structures of Fe_3_Sn_2_ and CoSn is depicted in Figure 1a. Structurally, these two materials differ in that CoSn contains a kagome monolayer (Co_3_Sn) separated by a Sn_2_ layer, while Fe_3_Sn_2_ contains a kagome bilayer (Fe_3_Sn/Fe_3_Sn) between each Sn_2_ layer. Throughout this work, we focus on three superlattices with differing layer thicknesses, which we denote as follows: **SL#1**: Fe­(6.5 nm)/[Fe_3_Sn_2_(3.9 nm)/CoSn­(5.6 nm)]_×4_/CaF_2_(2.9 nm), **SL#2**: Fe­(6.9 nm)/[Fe_3_Sn_2_(6.0 nm)/CoSn­(6.3 nm)]_×3_/CaF_2_(3.0 nm), and **SL#3**: Fe­(8.5 nm)/[Fe_3_Sn_2_(2.1 nm)/CoSn­(9.6 nm)]_×4_. These thickness values are obtained from PNR fitting, which is a measure of average layer thickness over the lateral 10 × 10 mm^2^ area of the samples. The CaF_2_ capping layer in SL#1 and SL#2 was deposited on the kagome layers with the aim of minimizing sample oxidation.

**Figure 1:**
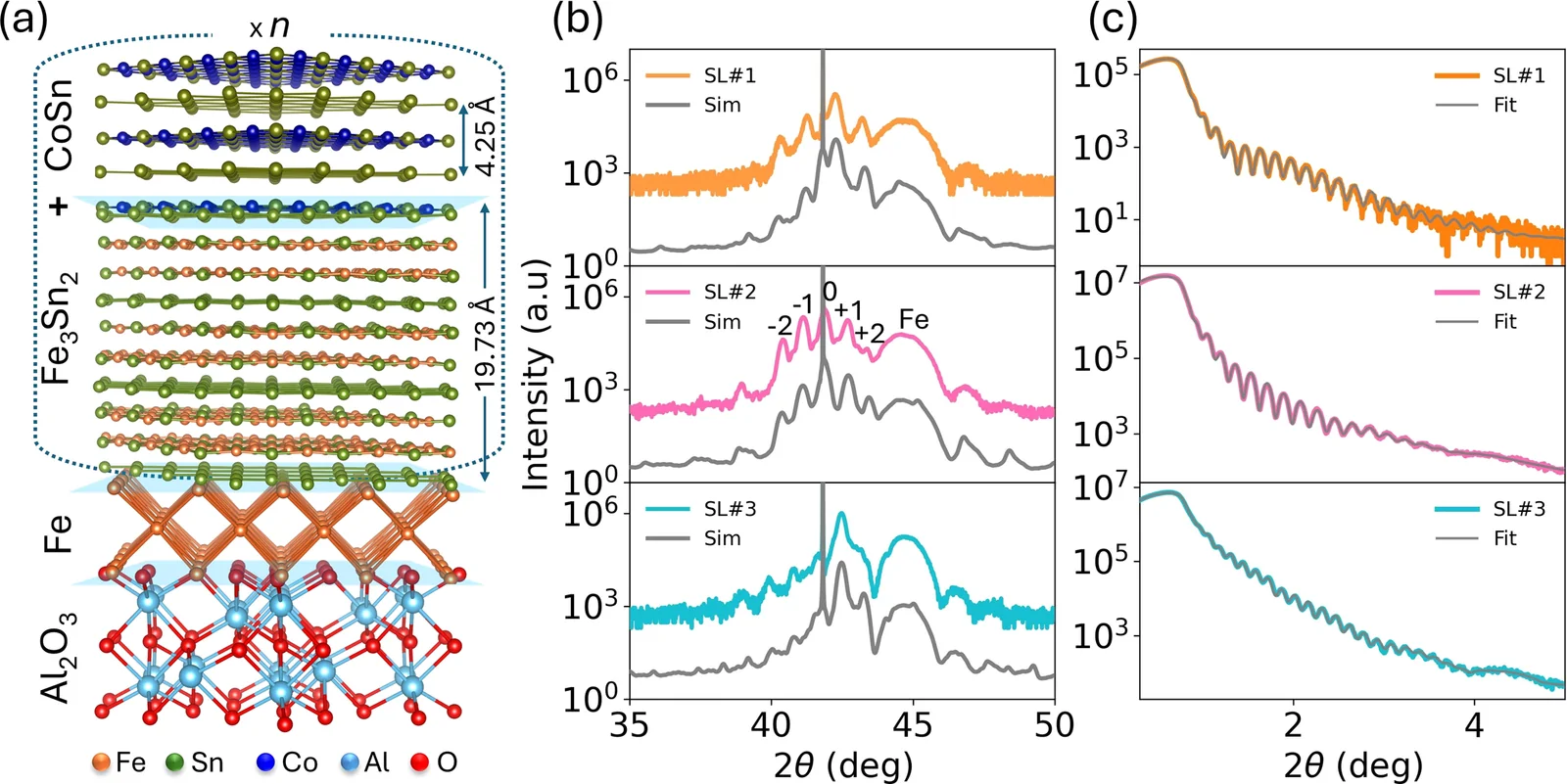
(a) Schematic of an Al_2_O_3_/Fe/[Fe_3_Sn_2_/CoSn]_×*n*_ superlattice; the arrows indicate the *c*-axis parameters of Fe_3_Sn_2_ and CoSn. (b) A zoomed-in version of high-resolution out-of-plane X-ray diffraction patterns from three superlattice films, along with corresponding simulations (gray). These simulated patterns are obtained after Gaussian smoothing (σ ≈ 7°) and an example of an unsmoothed pattern is presented in the Supporting Information. The main (0) and satellite peaks are marked with (±1, ±2) in the middle panel. The “0” reflection corresponds to the combined intensity from the 0009 Fe_3_Sn_2_ and 0002 CoSn peaks. Experimental patterns are shifted vertically for visual comparison with simulation. (c) X-ray reflectivity (displayed in intensity) of the superlattices, along with corresponding fits in gray.

High-resolution out-of-plane X-ray diffraction (XRD) patterns obtained from these samples, shown in Figure 1b, exhibit well-defined peaks arising from the average *c*-axis parameter of the Fe_3_Sn_2_ and CoSn layers (indicated by “0” in Figure 1b) as well as satellite peaks due to the superstructure (“±1”, “±2”). Here, we note that the satellite peaks arise from the significant structural differences between the Fe_3_Sn_2_ and CoSn layers, despite the minimal X-ray scattering contrast between Fe and Co. By comparing the measured data with 000*L* XRD simulations, we were able to estimate *c*-axis lattice parameters and thicknesses of the individual layers. For Fe_3_Sn_2_, we obtained *c*-axis parameter values between 19.6 and 19.7 Å, in agreement with bulk materials.^32^ Similarly, the *c*-axis parameters (≈4.25 Å) of CoSn layers are consistent with bulk values.^33,34^ Diffraction scans carried out over a wider angular range (shown in the Supporting Information) contain only the 110 Fe and 000*l* kagome reflections, confirming that the kagome layers are *c*-axis oriented and phase pure. X-ray reflectometry (XRR) measurements were carried out to quantify the surface and interfacial roughness and the individual layer thicknesses. Clear thickness fringes are visible in the data. However, given the minimal differences in X-ray scattering length density between Fe_3_Sn_2_ and CoSn, the XRR does not show clear features from the superlattice periodicity. The reflectivity data and best fits, utilizing the thicknesses obtained from the XRD analysis to inform the allowed range of fitting parameters, are shown together in Figure 1c. The total sample thicknesses obtained from XRR and XRD, presented in the Supporting Information, are in good agreement. Note that the obtained XRD thickness values, by the nature of the diffraction modeling, are constrained to be integer multiples of monolayer thicknesses.

The interfacial structure was further characterized using high-angle annular dark-field (HAADF) scanning transmission electron microscopy combined with energy-dispersive X-ray spectroscopy. Figure 2a shows an elementally resolved EDS map of SL#1, with distinct layers of Fe, Fe_3_Sn_2_, CoSn, and CaF_2_ clearly visible between well-defined interfaces. While the superlattices are predominantly well-ordered, the STEM-EDS imaging reveals regions of local structural mosaicity and lateral chemical fluctuations, examples of which are shown in the Supporting Information. A high-resolution cross-sectional STEM image is presented in Figure 2b, highlighting a single Fe_3_Sn_2_/CoSn interface with the different kagome stacking in the respective layers. Within the Fe-containing layer, there appears to be a single Fe_3_Sn kagome monolayer immediately at the interface, indicating that local deviations in the stacking sequence can be present within Fe_3_Sn_2_ and may be more likely to form at the interface. The elemental depth profile obtained from EDS mapping is presented in Figure 2c, showing the modulation of distinct layers. The average thickness of each Fe_3_Sn_2_ and CoSn layer obtained from STEM-EDS is 3.6 and 6.6 nm, respectively, in good agreement with the thicknesses obtained from XRR (2.9 and 6.5 nm). Together, our XRD and STEM measurements confirm the successful growth of smooth superlattices with clear interfaces separating the two distinct kagome metals.

**Figure 2:**
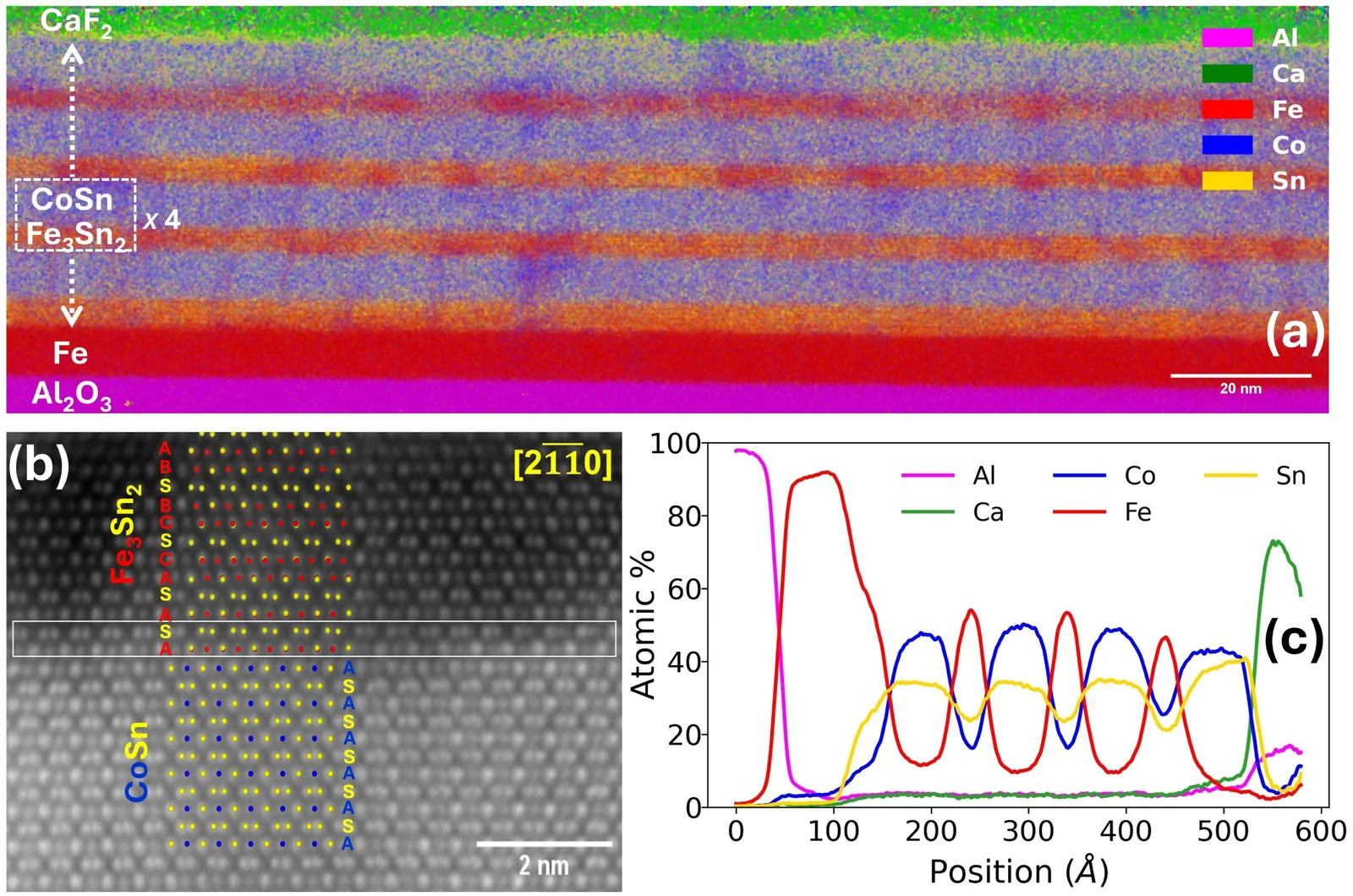
(a) STEM-EDS map of SL#1. (b) HAADF-STEM image of a selected Fe_3_Sn_2_/CoSn interface. The white rectangular box highlights the interface, while the red, blue, and yellow dots indicate Fe, Co, and Sn atomic columns. (c) Compositional depth profile for selected elements from the EDS image in (a), where the composition is normalized to 100%.

The layer-resolved magnetization in the superlattices was probed using specular, non-spin-flip PNR carried out at room temperature in an applied magnetic field of 0.4 T. Shown in Figure 3 (top rows), the spin-up (R^+^) and spin down (R^–^) reflectivity curves exhibit significant splitting due to the net magnetization of each sample. The experimental curves were fitted with models in which layer thickness, layer magnetization, interfacial chemical and magnetic roughness, and nuclear scattering length densities (SLDs) were allowed to vary. The best fit for each superlattice is shown in the top rows of Figure 3. Spin asymmetry, defined as (R^+^ – R^–^)/(R^+^ + R^–^), is plotted in the middle rows of Figure 3 for all superlattices, along with the fitted curves. The nuclear and magnetic SLD depth profiles obtained from the fits are shown in the bottom panels of Figure 3. The obtained fitted parameters are given in Table 1, while additional details about the PNR fits and comparisons between other models are presented in Supporting Information. The nuclear SLD values of each layer are within 5% of the theoretical values, which are 5.02 × 10^–6^ Å^–2^ and 2.60 × 10^–6^ Å^–2^ for Fe_3_Sn_2_ and CoSn, respectively. Due to the large difference in the nuclear SLD of Co compared to Fe, neutron reflectivity is significantly more sensitive to the superlattice structure compared to XRR, making PNR a powerful tool to probe structural information as well as magnetic information in these samples. This large nuclear scattering contrast between Fe_3_Sn_2_ and CoSn can be seen in Figure 3 (bottom, red lines). The layer thicknesses obtained from PNR are in excellent agreement with those obtained from XRD, XRR, and STEM-EDS, with a full comparison given in the Supporting Information. Note that in the models, the fitting parameters for the Fe_3_Sn_2_ layers are constrained to be identical, with the exception of the bottom Fe_3_Sn_2_ layer deposited on Fe and the topmost CoSn layer. The average chemical roughness at the Fe_3_Sn_2_/CoSn interfaces is 1.0 ± 0.13 nm, 1.2 ± 0.08 nm, and 0.3 ± 0.16 nm in SL#1, SL#2, and SL#3, respectively—values that are less than the *c*-axis parameter of Fe_3_Sn_2_. These values, as well as the bulk-like nuclear SLD values obtained for each layer, indicate that chemical intermixing is limited to the near-interfacial regions.

**Table 1:** Fitted Parameters (95% Interval) from PNR Data and the Converted Moment^a^

	SL#1			SL#2			SL#3		
	*t* (nm)	SLD_M_/10^–6^ Å^–2^	μ_B_/fu	*t* (nm)	SLD_M_/10^–6^ Å^–2^	μ_B_/fu	*t* (nm)	SLD_M_/10^–6^ Å^–2^	μ_B_/fu
Fe(1)	6.65(±0.03)	5.21(±0.03)	2.27(±0.01)	6.95(±0.03)	5.29(±0.01)	2.31(±0.01)	1.10(±0.04)	5.39(±0.01)	2.35(±0.01)
Fe(2)							7.56(±0.06)	5.39(±0.01)	2.35(±0.01)
Fe_3_Sn_2_ (bot)	4.32(±0.06)	0.99(±0.05)	2.98(±0.16)	5.80(±0.05)	1.0(±0.06)	3.01(±0.18)	1.48(±0.06)	0.99(±0.12)	2.98(±0.07)
CoSn (bulk)	5.58(±0.07)	0.0	0.0	6.38(±0.06)	0.0	0.0	9.39(±0.07)	0.0	0.0
Fe_3_Sn_2_ (bulk)	3.95(±0.01)	1.49(±0.04)	4.49(±0.10)	6.01(±0.06)	0.87(±0.02)	2.62(±0.08)	2.24(±0.03)	1.30(±0.07)	3.91(±0.18)
CoSn (top1)							7.63(±0.11)	0.0	0.0
CoSn (top2)	6.79(±0.03)	0.15(±0.04)	0.18(±0.04)	6.65(±0.1)	0.18(±0.01)	0.22(±0.01)	2.81(±0.08)	0.65(±0.04)	0.80(±0.07)

a Only SL#3 has Fe, and the topmost CoSn has divided layers.

**Figure 3:**
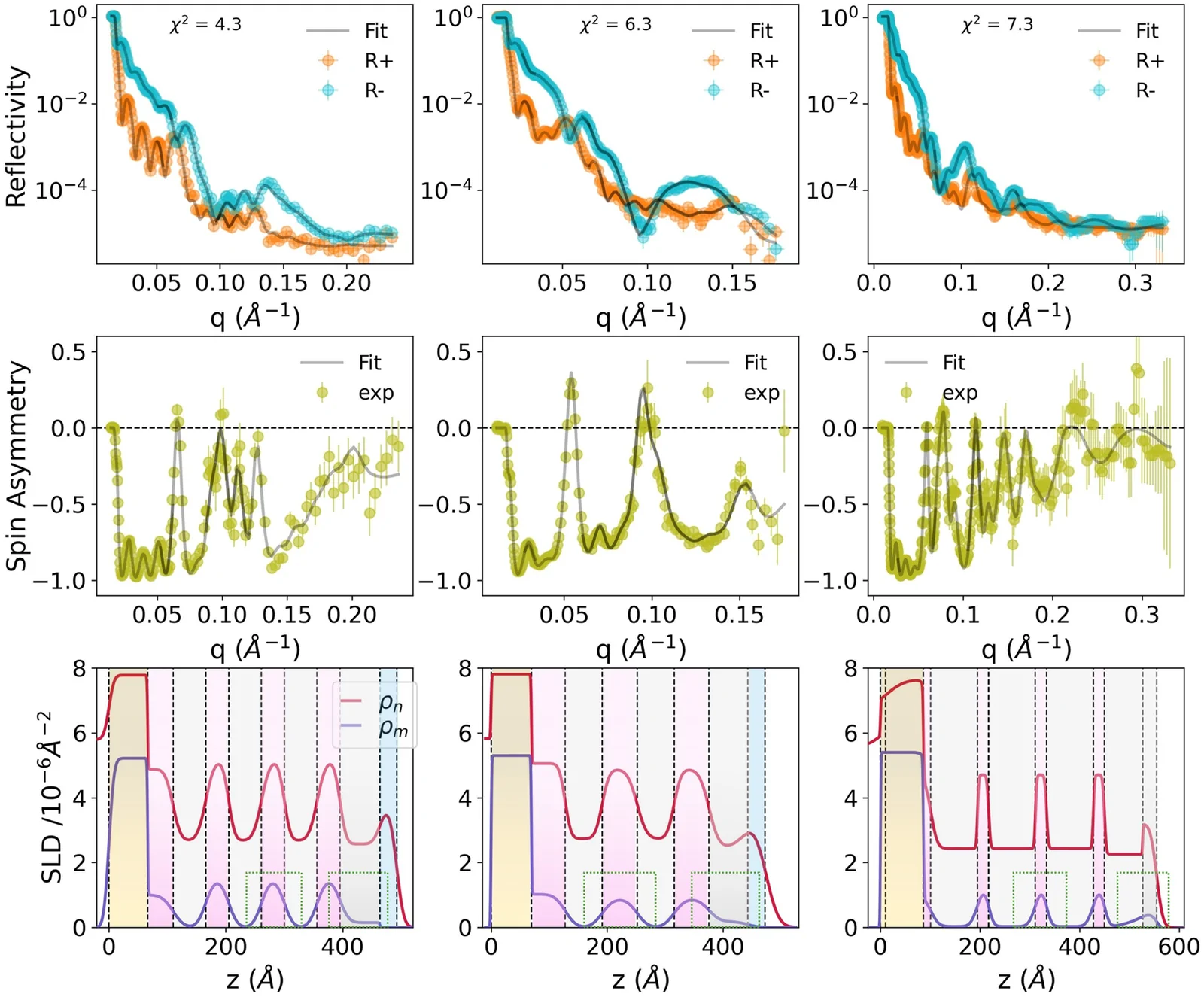
Polarized neutron reflectivity (top rows), spin asymmetry (middle rows), and SLD profiles (bottom rows) are shown for SL#1, SL#2, and SL#3. Corresponding χ^2^ values from data fitting are given in the top panel. Tan, pink, gray, and blue shaded boxes in the SLD profiles represent the Fe, Fe_3_Sn_2_, CoSn, and CaF_2_ layers in the superlattices, respectively. Green rectangular dotted boxes in the bottom panel represent the areas plotted in Figure 4a,b.

The PNR fits show the presence of magnetization in the Fe and Fe_3_Sn_2_ layers in all three superlattices, consistent with ferromagnetic order. The magnetization within the Fe_3_Sn_2_ layers is slightly reduced from the single-crystal bulk value (≈5.7 μ_B_/f.u.) with the best fits yielding ≈4.5 μ_B_/f.u., ≈2.6 μ_B_/f.u., and ≈3.9 μ_B_/f.u. in SL#1, SL#2, and SL#3, respectively. Most strikingly, the ferromagnetism is retained in the single-unit-cell limit in SL#3, indicating robust magnetic interactions even when the Fe_3_Sn_2_ layer thickness has been reduced to only three adjacent Fe_3_Sn bilayers (≈2 nm, equivalent to one unit cell along the *c*-axis). The magnetic profiles within the kagome superstructures largely follow the chemical profiles. Models that introduced magnetic dead layers within Fe_3_Sn_2_ or strongly magnetized interfacial CoSn layers were less successful in reproducing the experimental data. Within the best-fit models, the interfacial magnetic roughness was allowed to vary independently of the chemical roughness. The obtained magnetic roughnesses (σ_M_) are slightly larger than the chemical roughness (σ_N_) for all three superlattices. The difference between the magnetic and chemical roughness, equal to (σ_M_) – (σ_N_), is 0.2 nm, 0.3 nm, and 0.7 nm for SL#1, SL#2, and SL#3, respectively. This points to the absence of interfacial dead layers in Fe_3_Sn_2_ and indicates that any local net ferromagnetism within CoSn is limited to approximately one-half of a unit cell in depth.

To better visualize the magnetic profile within the confined Fe_3_Sn_2_, Figure 4a presents the magnetization profile for a representative Fe_3_Sn_2_ layer from each superlattice. We have converted the magnetic SLD into volume magnetization using the equation: SLD_M_ = *C* × *m*, where *m* is the magnetic moment (emu) per volume and *C* = 2.853 × 10^–9^ cm^3^ Å^–2^ emu^–1^ and finally to kA/m. Within this set of superlattices, the magnetization does not exhibit a clear dependence on the Fe_3_Sn_2_ layer thickness. From this, we conclude that the variation in the magnitude of magnetization between superlattices is likely driven by extrinsic factors, such as subtle differences in Fe:Sn stoichiometry. Given that antiferromagnetic FeSn and ferromagnetic Fe_3_Sn_2_ differ only in their stacking sequence (Fe_3_Sn/Sn_2_/Fe_3_Sn/Sn_2_ vs Fe_3_Sn/Fe_3_Sn/Sn_2_), the presence of excess Sn could be accommodated by the formation of Fe_3_Sn monolayers instead of Fe_3_Sn/Fe_3_Sn bilayers. The local presence of Fe_3_Sn monolayers instead of Fe_3_Sn/Fe_3_Sn bilayers would reduce the local Fe atomic density, thus diminishing the local magnetization. Future structural studies, beyond the scope of this work, would be useful to establish the relationship between Fe_3_Sn_2_ off-stoichiometry, stacking faults, and magnetization.

**Figure 4:**
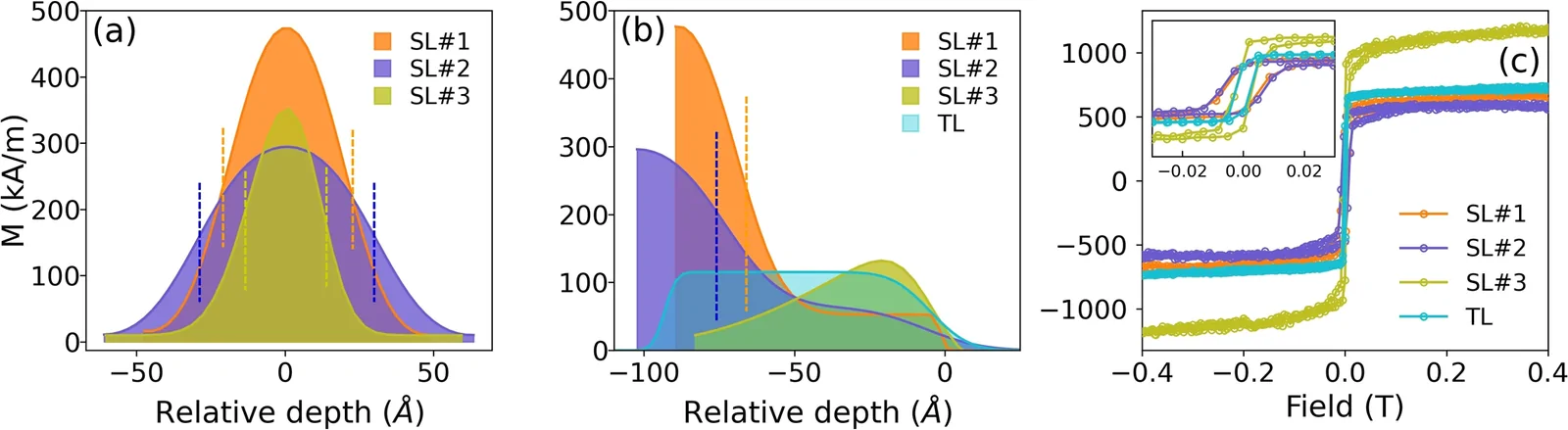
(a) Magnetic profile of a representative Fe_3_Sn_2_ layer for each of the three superlattices. (b) Magnetic profile at the surface of the superlattices and trilayer sample. The dotted lines in (a,b) indicate the CoSn/Fe_3_Sn_2_ and Fe_3_Sn_2_/CoSn interfaces within the heterostructures. In (b), 0 Å depth on the *x*-axis indicates the surface of the sample in SL#3 and TL, and the interface of CoSn and CaF_2_ in SL#1 and SL#2. (c) Total volume magnetization of the three superlattices and the trilayer as a function of the applied magnetic field at 300 K.

A second striking result is that the surface-most CoSn layer in all three superlattices shows a non-negligible magnetic SLD. This is in contrast to the nonsurface CoSn, which exhibits essentially no magnetization in the center of the layers, consistent with the paramagnetic nature of bulk CoSn. We note that ferromagnetism is not present at any composition across the Co_1–*x*_Fe_*x*_Sn phase diagram,^35^ indicating that even in the case of Fe substitution into CoSn, for instance, due to chemical intermixing across CoSn/Fe_3_Sn_2_ interfaces, a net magnetization would not be expected in CoSn. Comparative PNR fits with and without surface magnetization are presented in the Supporting Information. We have also carried out depth-profiling X-ray photoemission spectroscopy measurements (see the Supporting Information), which confirm the absence of Fe at the surface.

To further verify the surface magnetization in CoSn, we synthesized a Fe­(8.5 nm)/Fe_3_Sn_2_(14 nm)/CoSn­(15 nm) trilayer (TL) sample and measured it under the same PNR conditions as the superlattices. Our best fit to the TL sample yields a similar magnetization in the surface region (≈ 8 nm) of the CoSn layer and no magnetization in the bulk region (see the Supporting Information for X-ray characterization and the PNR fits). This result highlights the power of PNR, as, without the depth-specific magnetic information, the surface magnetization in CoSn would not have been identified. The magnetic depth profile of the near-surface region for the three superlattices and the trilayer is plotted together in Figure 4b to summarize the surface magnetization found in CoSn across these samples. The magnetization is larger in the uncapped samples, 0.80 μ_B_/f.u. in SL#3 and 0.40 μ_B_/f.u. in TL, compared to the capped samples, 0.18 μ_B_/f.u. and 0.22 μ_B_/f.u. in SL#1 and SL#2. Since bulk CoSn is paramagnetic in nature, we attribute this behavior to oxidation at the top of the CoSn layer. It has been reported in FeSn kagome metal that Sn is more prone to oxidation in the stanene layer than Fe in the Fe_3_Sn kagome layer.^36^ Another report found that Co-substituted SnO_2_ thin films exhibit ferromagnetic ordering with a moment of 0.47 μ_B_/Co at room temperature.^37^ Therefore, we hypothesize that Co is segregated and/or substituted in a Sn oxide layer (stanene), resulting in a net moment coming from Co.

Magnetometry measurements were performed on all samples at 300 K with an in-plane magnetic field. These results are presented in Figure 4c. The measured magnetization was normalized by the summed volume of the Fe, Fe_3_Sn_2_, and surface CoSn layers. The volume magnetization data of all samples saturates at approximately ≈10 mT, confirming that the magnetization is in a saturated state during the PNR measurements. SL#3 exhibits the largest saturation magnetization because the Fe buffer layer is slightly thicker in this sample compared to the others, as can be seen from the area of the SLD profile in Figure 3. While the Fe buffer thickness in SL#1 and SL#2 is equal, the volume magnetization of SL#1 is larger than that of SL#2, consistent with the PNR result showing larger magnetization in the Fe_3_Sn_2_ within SL#1 compared to SL#2. We present a comparison between the volume magnetization obtained from magnetometry and the average volume magnetization from PNR measurements in the Supporting Information, highlighting the excellent quantitative agreement between the two measurement techniques.

These results establish a synthesis strategy for realizing confined magnetic kagome layers, with controlled layer thickness, embedded in similarly structured nonmagnetic kagome metals. Dimensionality and finite thickness have been important themes in kagome research.^28,38–43^ However, in ultrathin monolithic films or flakes, intrinsic physical effects are inevitably convoluted with surface oxidation (impacting the top few nanometers of kagome metals) or interfacial defects arising between a crystallographically dissimilar film and substrate. In contrast, the superlattice approach presented here provides a clean platform for understanding thickness-dependent phenomena in these materials. Our observation that magnetic ordering is retained in a single unit cell of Fe_3_Sn_2_ without interfacial dead layers establishes this kagome metal as being well-suited to serve in model structures designed to understand the evolution of topological or anomalous magnetotransport responses as a function of thickness or layer stacking. The presence of magnetization at the surface of CoSn further highlights the importance of mitigating extrinsic sources of material response in studying the intrinsic properties of kagome metals. In the specific case of CoSn, the discovery of surface ferromagnetism has important implications for studies of magnetotransport properties or the use of spectroscopy techniques that are surface-sensitive (e.g., photoemission, X-ray dichroism, optical probes). Unless performed after cleavage in a vacuum, these measurements may produce magnetic responses that are absent from the bulk of CoSn and not representative of its intrinsic properties.

## Conclusions

In this work, we have successfully grown high-quality kagome superlattices consisting of ferromagnetic Fe_3_Sn_2_ and paramagnetic CoSn layers on Fe-buffered Al_2_O_3_(0001) substrates using MBE. To the best of our knowledge, this is the first time superlattices consisting of kagome magnets with different transition metal chemistries have been synthesized. The combination of XRD and STEM-EDS measurements evidences the phase purity of the individual layers and the presence of well-defined interfaces between layers. Ferromagnetism is retained in the Fe_3_Sn_2_ layers, even at the single unit cell (≈2 nm) level. The magnetization within the confined Fe_3_Sn_2_ layers extends slightly beyond the chemical profile of the Fe_3_Sn_2_/CoSn interfaces, confirming the absence of magnetic dead layers and suggesting the possibility of a very local induced magnetization within the interfacial CoSn unit cell. We have also discovered the presence of ferromagnetic magnetization in the surface CoSn layer, in contrast to its bulk paramagnetism. This work demonstrates the feasibility of epitaxial superlattices as a means to realize kagome metals in confined forms, thus enabling future studies of how finite thickness and coupling between adjacent kagome layers alter magnetotransport and other functional responses in this emerging family of topological materials.

## Methods

### Growth of Superlattices via Molecular Beam Epitaxy

The superlattices were grown using molecular beam epitaxy (Omicron-modified LAB-10 system) on (0001)-oriented Al_2_O_3_ substrates. Atomic fluxes were provided through the sublimation of Fe (99.95%, slug, Alfa Aesar), Co (99.95%, shot, Alfa Aesar), CaF_2_ (pieces from single crystal substrates), and the evaporation of Sn (99.99%, shot, Alfa Aesar) from effusion cells (MBE Komponenten). The relative flux from each effusion cell was measured with a quartz crystal microbalance immediately prior to deposition; these relative flux values were converted to absolute flux using X-ray reflectivity and Rutherford backscattering spectrometry (RBS) measurements of calibration depositions. Shutter times and flux rates were set to control the composition and thickness of all layers. Prior to deposition, the substrates were sonicated in an acetone bath for 15 min, loaded into the chamber, and heated to a temperature of ≈425 °C predeposition. The Fe buffer layer was deposited at ≈400 °C, while the Fe_3_Sn_2_ and CoSn layers were grown alternately using codeposition at ≈450 °C. While bulk Fe_3_Sn_2_ is only the equilibrium phase above 610 °C,^44^ we find that phase-pure Fe_3_Sn_2_ films can be grown by codeposition at lower temperatures, consistent with other reports.^25,31,45^ SL#1 and SL#2 were capped with a thin layer (≈3 nm) of insulating CaF_2_, deposited while the sample cooled following superlattice growth, which appears to decrease, but not prevent, surface oxidation of the kagome films.

### X-ray Scattering

X-ray diffraction (XRD) and reflectivity (XRR) were measured using a Rigaku SmartLab diffractometer with a Ge (220) double-bounce monochromator and Cu K_α1_ radiation (λ = 1.5406 Å). XRR fitting and XRD simulations were performed with the GenX software program.^46^ For XRR fitting, we used the model of layer sequences as follows: Al_2_O_3_/Fe/Fe_3_Sn_2_(bot)/[CoSn/Fe_3_Sn_2_]_*n*_/CoSn­(top)/CaF_2_ (in SL#1,2 only). The Fe_3_Sn_2_ and CoSn layers within the [CoSn/Fe_3_Sn_2_]_*n*_ bulk superlattice region were constrained to have the same thicknesses, densities, and roughness. Meanwhile, for the XRD simulation, we used a layer sequence of Al_2_O_3_/Fe/[Fe_3_Sn_2_/CoSn]_*n*_/CaF_2_ (in SL#1,2), in which atomic positions are assigned within a unit cell in each different material based on the bulk crystal structures. The out-of-plane lattice parameter and the number of unit cells were varied to best reproduce the experimental data. All Fe_3_Sn_2_ layers were constrained to have the same atomic positions and *c*-axis parameter, as were the CoSn layers. No interfacial roughness was included within the XRD simulation. Note that the obtained XRD thickness values, by the nature of the diffraction modeling, are constrained to be integer multiples of monolayer thicknesses. RBS was carried out at Rutgers University, within the Laboratory for Surface Modification, and analyzed using the SIMNRA software package.

### Transmission Electron Microscopy

Cross-sectional TEM specimens were prepared using a Thermo Fisher Helios 5 focused ion beam with a Ga^+^ ion beam of 30 and 8 keV, followed by a final milling step at 2 keV to reduce damage. To protect the surface from ion beam damage, carbon and tungsten layers were deposited before milling. HAADF-STEM images were acquired using a Cs-corrected Thermo Fisher Scientific Spectra operated at 200 kV, with a beam semiconvergence angle of 25 mrad. The energy-dispersive X-ray spectroscopy (EDS) mapping was acquired with a Thermo Fisher Scientific Spectra operated at 120 kV, equipped with a Dual-X EDS detector. The STEM-EDS maps were acquired with a probe current of 215 pA and a semiconvergence angle of 25 mrad, continuously raster-scanned with drift correction and a dwell time of 5 μs, resulting in a total of ∼9 million X-ray counts. Elemental maps and atomic percent quantification were obtained by background subtraction and peak fitting, followed by standardless Cliff–Lorimer quantification.

### Magnetometry Measurements

Thin-film in-plane DC magnetometry was carried out using a Physical Properties Measurement System (Quantum Design) with a vibrating sample magnetometry (VSM) module.

### Polarized Neutron Reflectometry and Simulations

Polarized neutron reflectometry measurements on SL#2 and SL#3 were performed at the MAGREF beamline at the Spallation Neutron Source, Oak Ridge National Laboratory. The SL#1 sample was measured at the AMOR beamline within the SINQ facility at the Paul Scherrer Institut. All the measurements were carried out at 300 K with an in-plane applied magnetic field of 0.4 T. The magnetic field ensures that the magnetization within the samples is saturated. As such, the scattered neutrons were collected without polarization analysis, yielding only spin-up (R^+^) and spin-down (R^–^) reflectivities. All samples were 1 cm^2^. Reflectivity curves were fit using the Refl1D software package.^47^ For PNR fitting, we used a similar model of layer sequences as follows: Al_2_O_3_/Fe/Fe_3_Sn_2_(bot)/[CoSn/Fe_3_Sn_2_]_×*n*_/CoSn­(top)/CaF_2_ (in SL#1 and SL#2 only). Similar to the XRR fitting approach, the Fe_3_Sn_2_ and CoSn layers within the [CoSn/Fe_3_Sn_2_]_*n*_ bulk superlattice region were constrained to have the same thicknesses, densities, and roughness.

## Supplementary Material



## Data Availability

The data used to make the graphs in this paper is available at DOI: 10.5281/zenodo.21511646.

## Notes

Detailed structural characterization of superlattice and trilayer films using XRD and XRR; detailed comparison of scattering length density profiles obtained from PNR and XRR fitting. It also includes alternative PNR fitting, a comparison of volume magnetization obtained from magnetometry and PNR measurements, X-ray photoemission data, and additional STEM-EDS images (PDF)
