# Supplementary material for: Confined Room-Temperature Ferromagnetism in Kagome (Fe3Sn2/CoSn) Superlattices
Source: ACS Nano. 2026 Aug 6;20(32):22530–8. doi: 10.1021/acsnano.6c03980 (PMC13492467; doi:10.1021/acsnano.6c03980)
Supplement: Supplementary file 1 [file nn6c03980_si_001.pdf]

Supporting Information
“Confined Room Temperature Ferromagnetism in Kagome (Fe_3Sn_2/CoSn) Superlattices”

Rajesh Dutta*, Christopher J. Jensen, Tarush Tandon, Prajwal M. Laxmeesha, Alexander Velic, Murat Tuna Pamuk, Chuqiao Shi, Yu-Tsun Shao, Timothy R. Charlton, Jochen Stahn, Alexander J. Grutter, Julie A. Borchers, and Steven J. May*

I | Structural characterization of superlattices and trilayer (TL) thin films

Figure S1a,b represent the side and top view of Fe_3Sn_2 and CoSn unit cells, respectively. In addition to the three superlattices, we grew a trilayer (TL) of Fe(8.5 nm)/Fe_3Sn_2(14 nm)/CoSn(15 nm) on a Al_2O_3 (0001) substrate to verify the surface CoSn magnetization. Wide angle XRD patterns between 10° - 70° in 2θ for all four samples are presented in Fig. S1c confirming phase purity and *c*-axis orientated growth. Fig. S1d and Fig. S1e present XRD and XRR measurements on the TL sample. Blue and red curves represent the simulated XRD patterns before and after applying Gaussian smoothing. The obtained thicknesses of superlattices and TL sample from XRD, XRR and PNR analysis are given in Table 1.

TABLE 1. Comparison of bulk Fe_3Sn_2 and CoSn layer thickness obtained from XRD, XRR and PNR fittings

SL#1

	XRD	XRR	PNR
Fe_3Sn_2	2.6 nm	3.9 nm	3.9 nm
CoSn	6.4 nm	5.7 nm	5.6 nm

SL#2

	XRD	XRR	PNR
Fe_3Sn_2	5.9 nm	5.6 nm	6.0 nm
CoSn	6.0 nm	6.8 nm	6.3 nm

SL#3

	XRD	XRR	PNR
Fe_3Sn_2	2.0 nm	1.8 nm	2.1 nm
CoSn	9.3 nm	9.6 nm	9.6 nm

TL

	XRD	XRR	PNR
Fe_3Sn_2	11.9 nm	13.0 nm	13.9 nm
CoSn	8.5 nm	14.9 nm	15.4 nm

II | Comparison of density depth profiles from polarized neutron reflectivity and X-ray reflectivity

Structural scattering length density (SLD) profiles from polarized neutron reflectivity (PNR) and X-ray reflectivity (XRR) fits are presented in Fig. S2 for each superlattice and TL. The top CoSn and bottom Fe_3Sn_2 layers were decoupled from remaining bulk SL to be fit independently in XRR fitting. The thicknesses presented in Table 1 from XRR fitting are averaged out between bulk and decoupled layers. The SLD profiles of the Fe_3Sn_2 and CoSn layers obtained from XRR and PNR fits are in good agreement.

III | Alternative PNR model fitting of SL#1, SL#2 and TL

Figure S3 presents PNR (R^*q^4), spin asymmetry (SA) and SLD depth profiles of SL#1 using a model where the magnetization within the center CoSn layers has been allowed to vary. The resultant fit yields a minimal difference between the SA obtained from this model and the model without bulk CoSn magnetization presented in the main text of the article replotted in top panel in Fig. S4. This result highlights how the PNR fits have limited sensitivity to a small magnetization within the center CoSn layers. A similar model has been tried in SL#2, but the fit did not take the bulk CoSn magnetization to be non-zero.

The PNR data was fit using models with and without surface magnetization in the topmost CoSn layer. For all four samples, fits wtih surface magnetization yielded the lowest chi-squared. Comparative models with and without surface magnetization are presented in Figs. S4, S5, and S6 for SL#1, SL#2, and TL, respectively. The models lacking surface magnetization fail to capture of subtle features at low-Q, which are highlighted with circles in the figures.

IV | Comparison of volume magnetization from PNR and magnetometry

Figure S7 presents a comparison between volume magnetization obtained from vibrating sample magnetometry (VSM) measurements and the PNR magnetic depth profiles. The average volume magnetization is obtained from PNR by converting the magnetic SLD into a volume magnetization (kA/m) and then integrating over the depth and finally normalizing by the total thickness of the Fe buffer, Fe_3Sn_2 and surface CoSn layers. For the VSM data, we have subtracted the diamagnetic signal from substrate and then divided with the same total volume (Fe buffer, Fe_3Sn_2 and surface CoSn layers) of the each sample. The two independent measurement techniques are in good agreement as the obtained saturation magnetization falls on the unity slope when plotted against each other.

V | X-ray photoemission spectroscopy

X-ray photoelectron spectroscopy (XPS) was performed with a PHI VersaProbe 5000 and Ar^+ sputtering for depth profiling. One survey scan and individual elemental spectra were collected presputter. Sputter time was set to 15 min with 30 s intervals and 30 s delayed acquisition time. XPS spectra and depth profiles were analyzed using CasaXPS software. Figure S8 presents the depth profiles of SL#2 and SL#3 samples and they don’t show any signal of Fe at the surface. This rules out Fe as the possible source of surface magnetization.

VI | HAADF-STEM imaging and EDS mapping

An example of structural domains is presented in Fig. S9, as revealed from HAADF-STEM imaging. Figs. S10 and S11 present STEM-EDS maps illustrating the presence of local chemical fluctuations within SL#1.

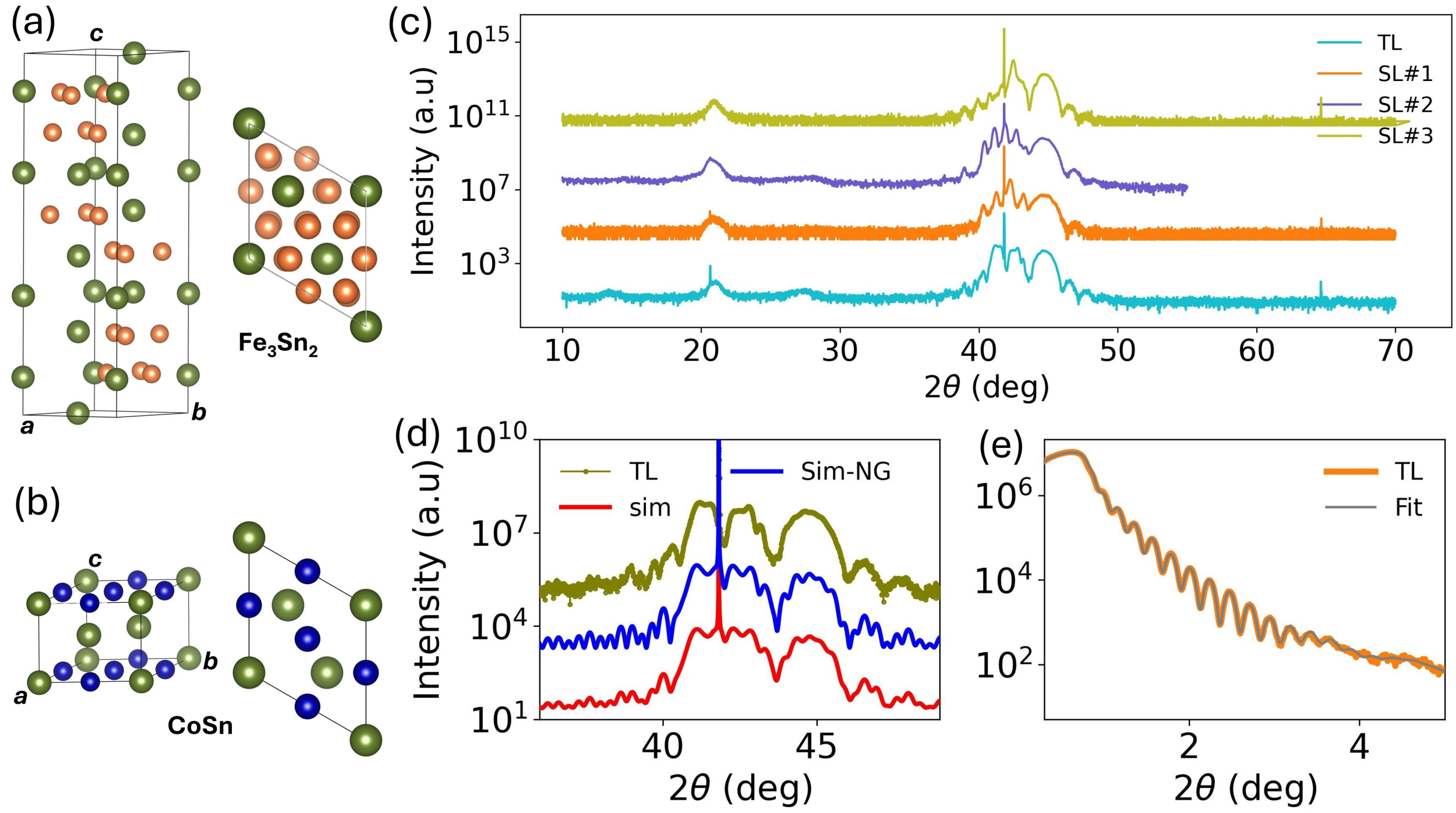

FIG. S1. Side and top view of the (a) Fe_3Sn_2 and (b) CoSn unit cells; green, blue, and orange spheres represent Sn, Co and Fe atoms, respectively. (c) Wide angle X-ray diffraction patterns of the superlattice and TL samples. (d) XRD data along with simulation for the TL sample. Red and blue curves indicated the XRD simulation with (sim) and without (sim-NG) Gaussian smoothing. (e) Measured XRR data and its fit for the TL sample.

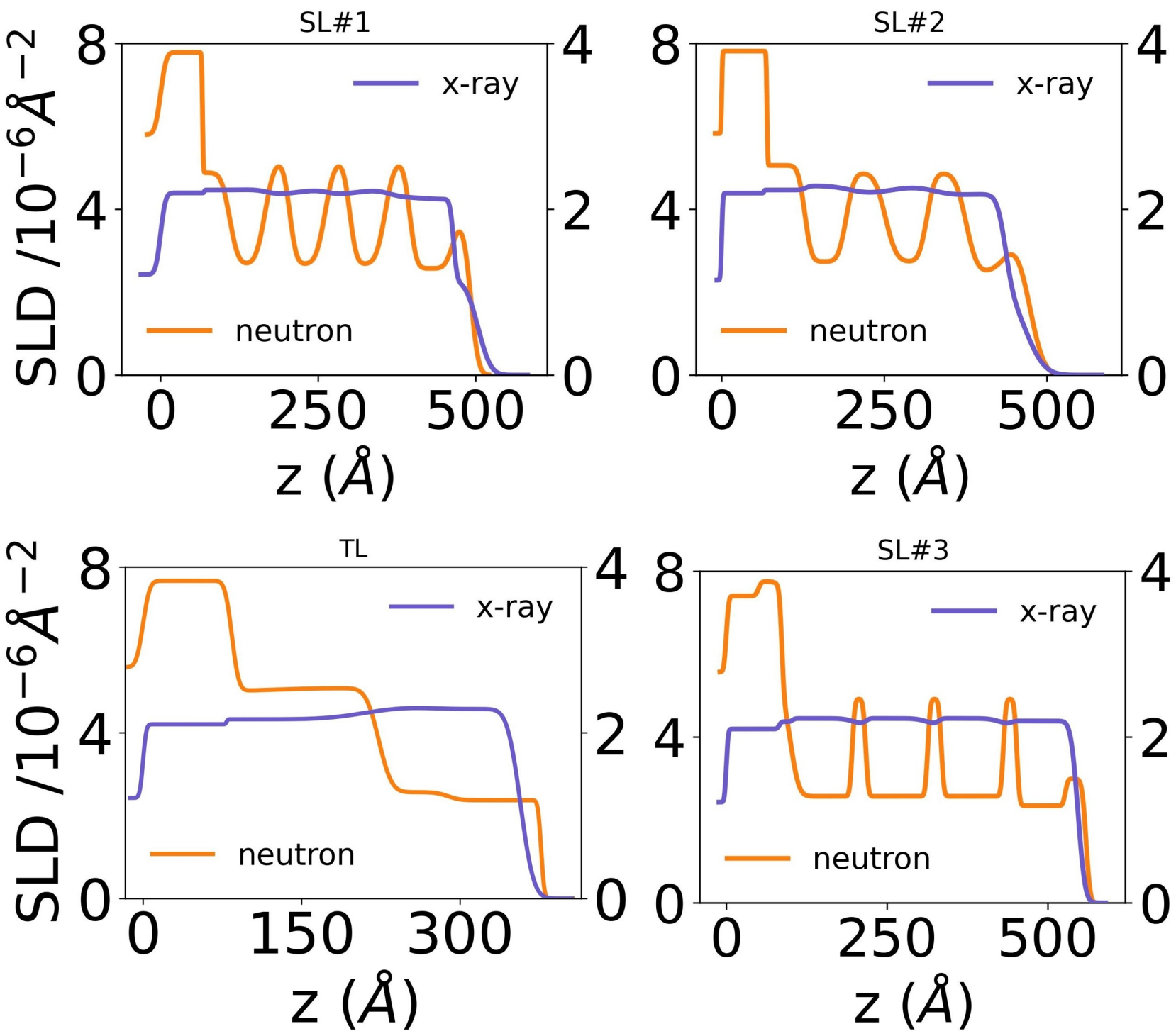

FIG. S2. Structural SLD profiles obtained from PNR and XRR for the superlattices and TL sample.

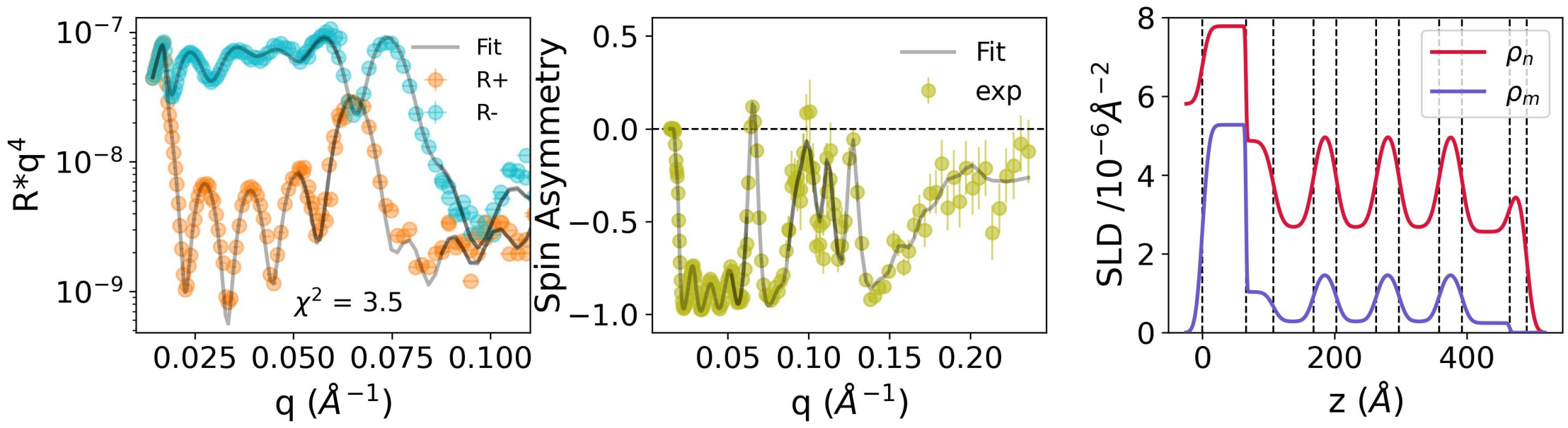

FIG. S3. Fitting results to an alternative PNR model on SL#1 in which there is a net magnetization within all CoSn layers.

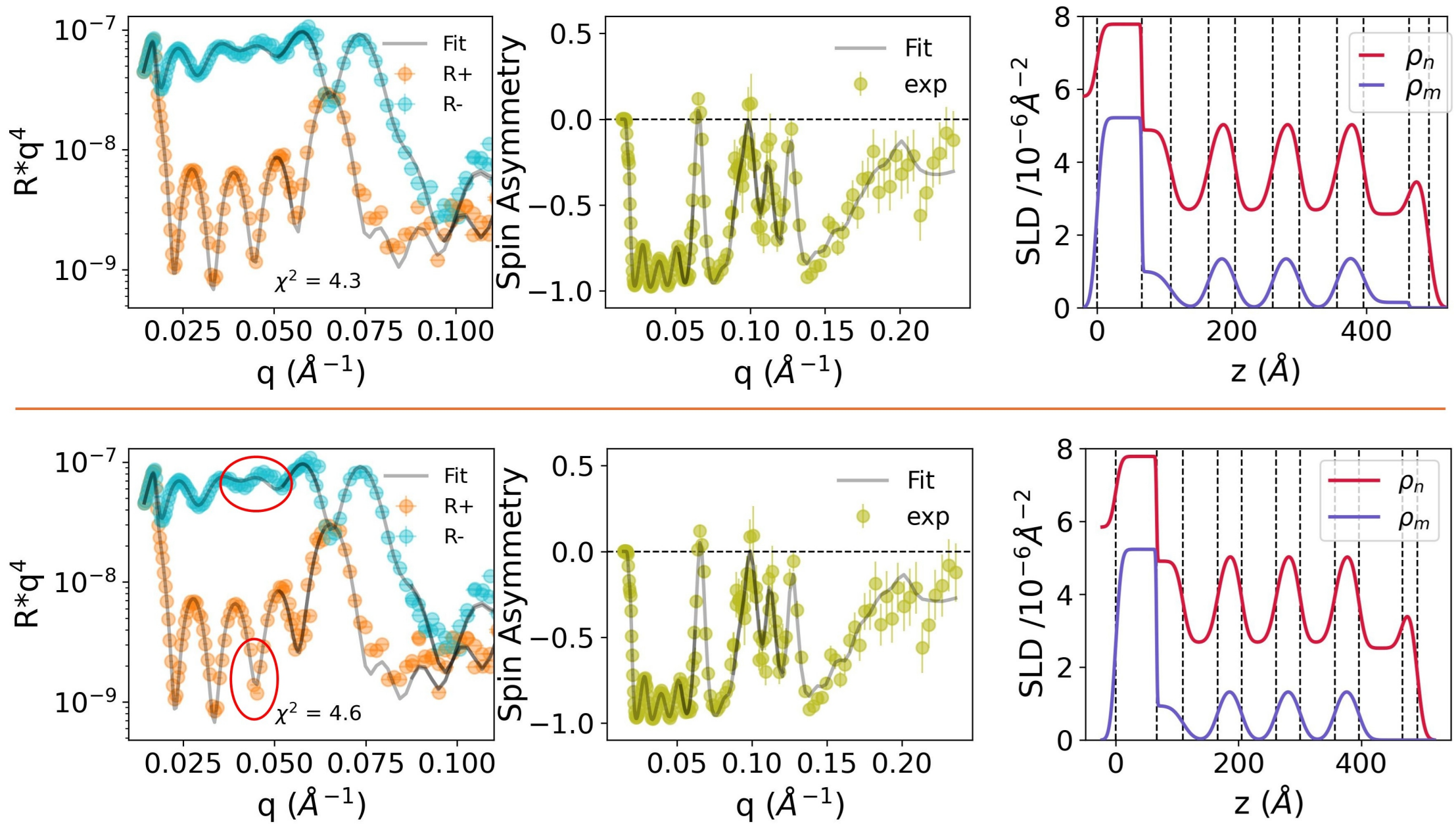

FIG. S4. PNR data fitting using two models of SL#1: with CoSn surface magnetization (top panel) and without CoSn surface magnetization (bottom panel). The fit in the top panel is the same as the main text.

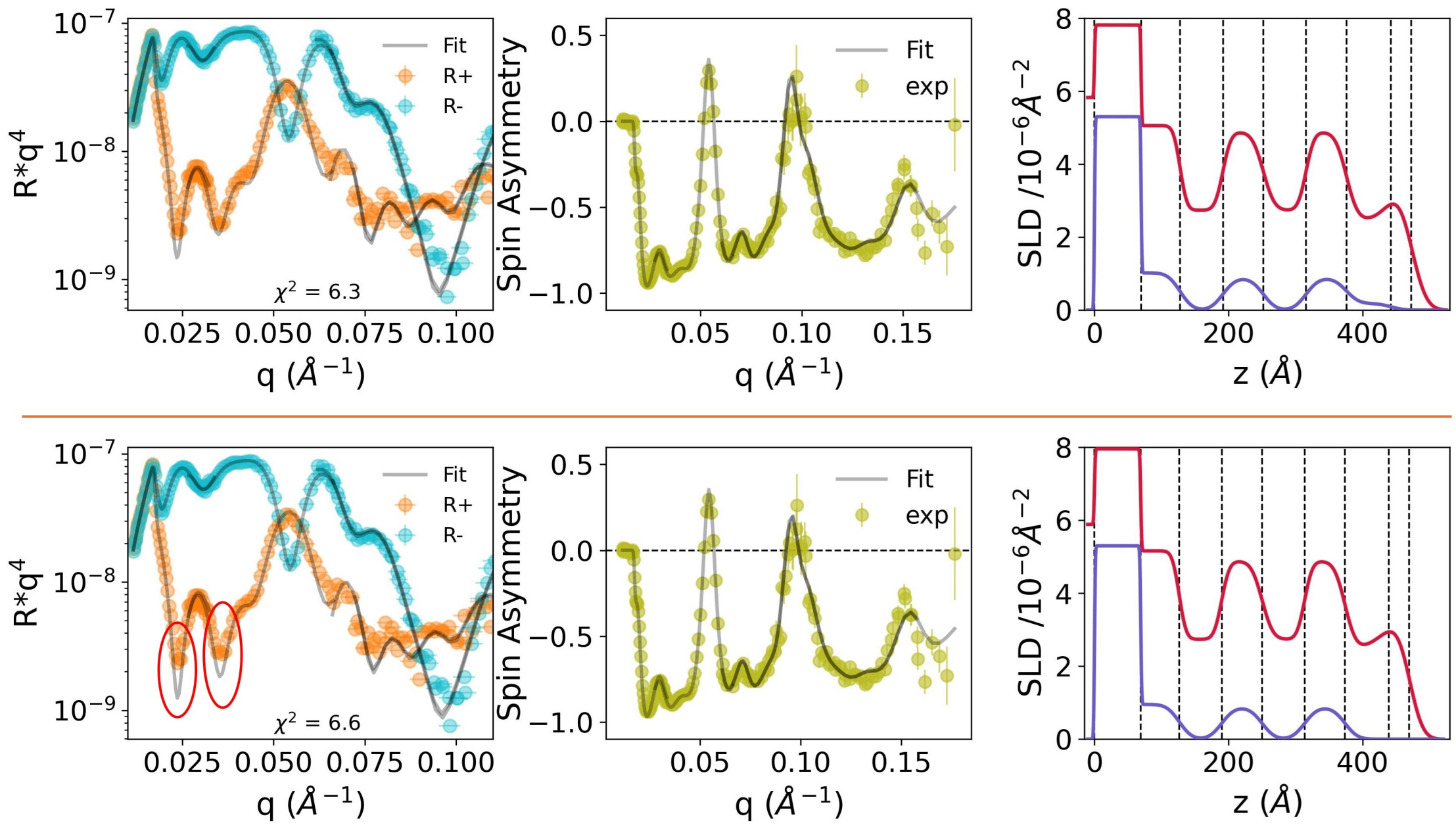

FIG. S5. PNR data fitting using two models of SL#2: with CoSn surface magnetization (top panel) and without CoSn surface magnetization (bottom panel). The fit in the top panel is the same as the main text.

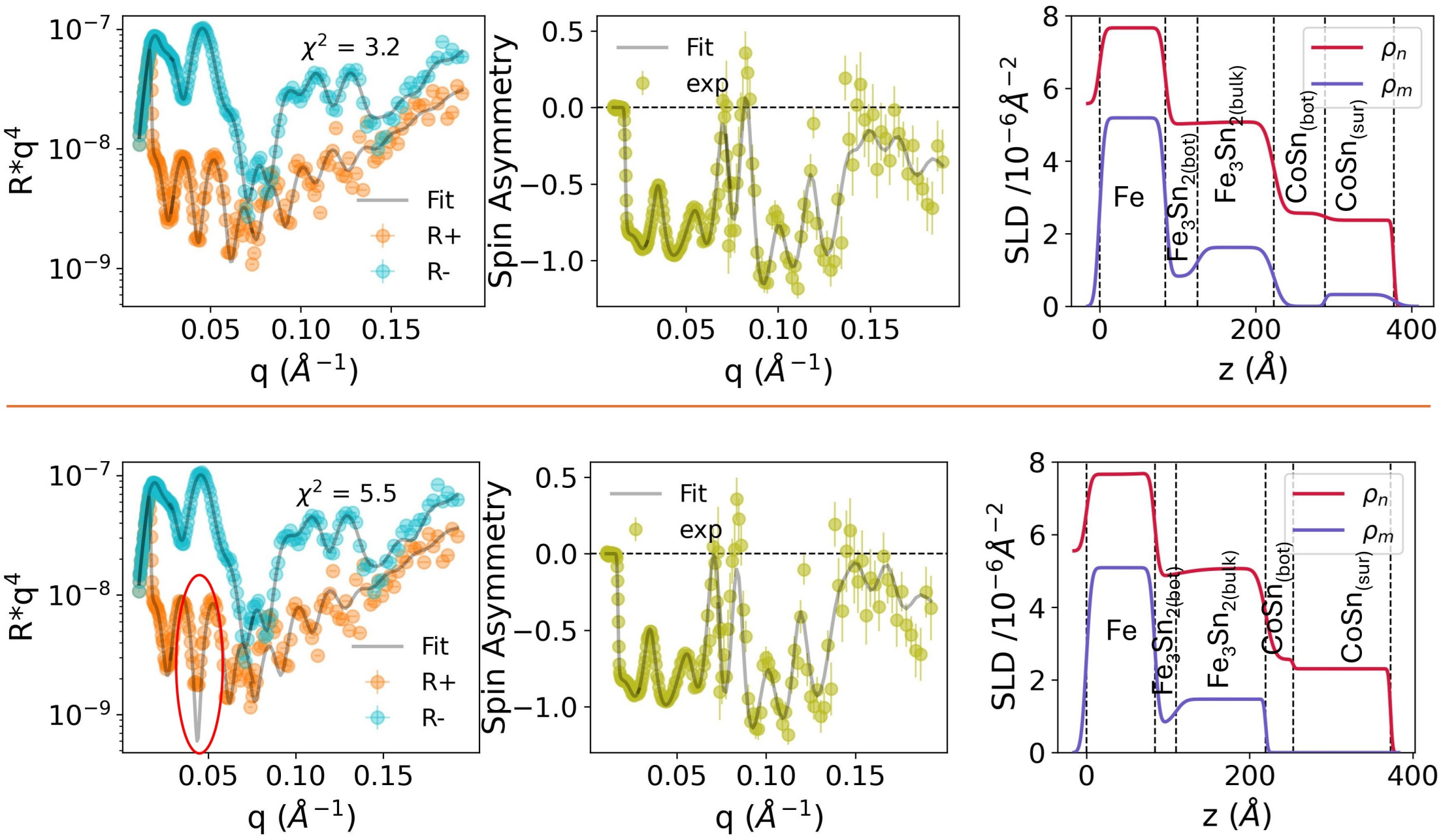

FIG. S6. PNR data fitting using two models of TL: with CoSn surface magnetization (top panel) and without CoSn surface magnetization (bottom panel).

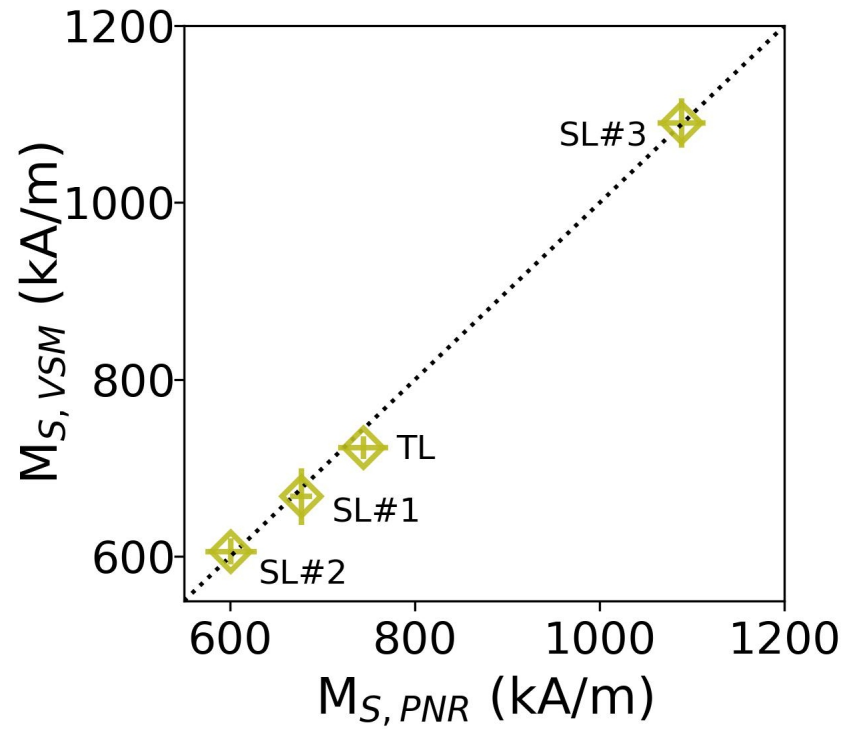

FIG. S7. Volume magnetization obtained from VSM and PNR measurements for superlattices and TL. Horizontal and vertical bars represent the error in volume magnetization corresponding to PNR and VSM data. For the VSM data, errors were calculated using a law of approach to saturation fit and for the PNR data, it was calculated using 95% interval values.

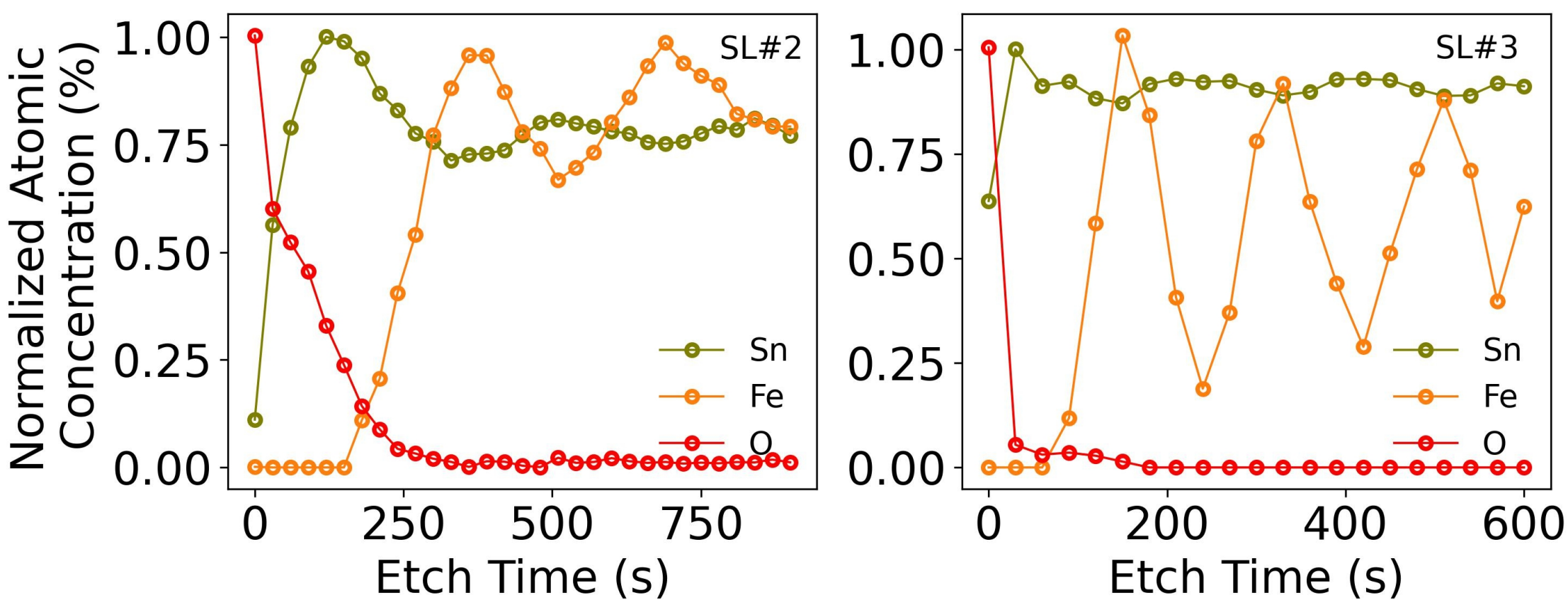

FIG. S8. XPS depth profiles of Sn, Fe and O from (left) SL#2 and (right) SL#3. The profiles are collected from the surface into the bulk of the SL layers.

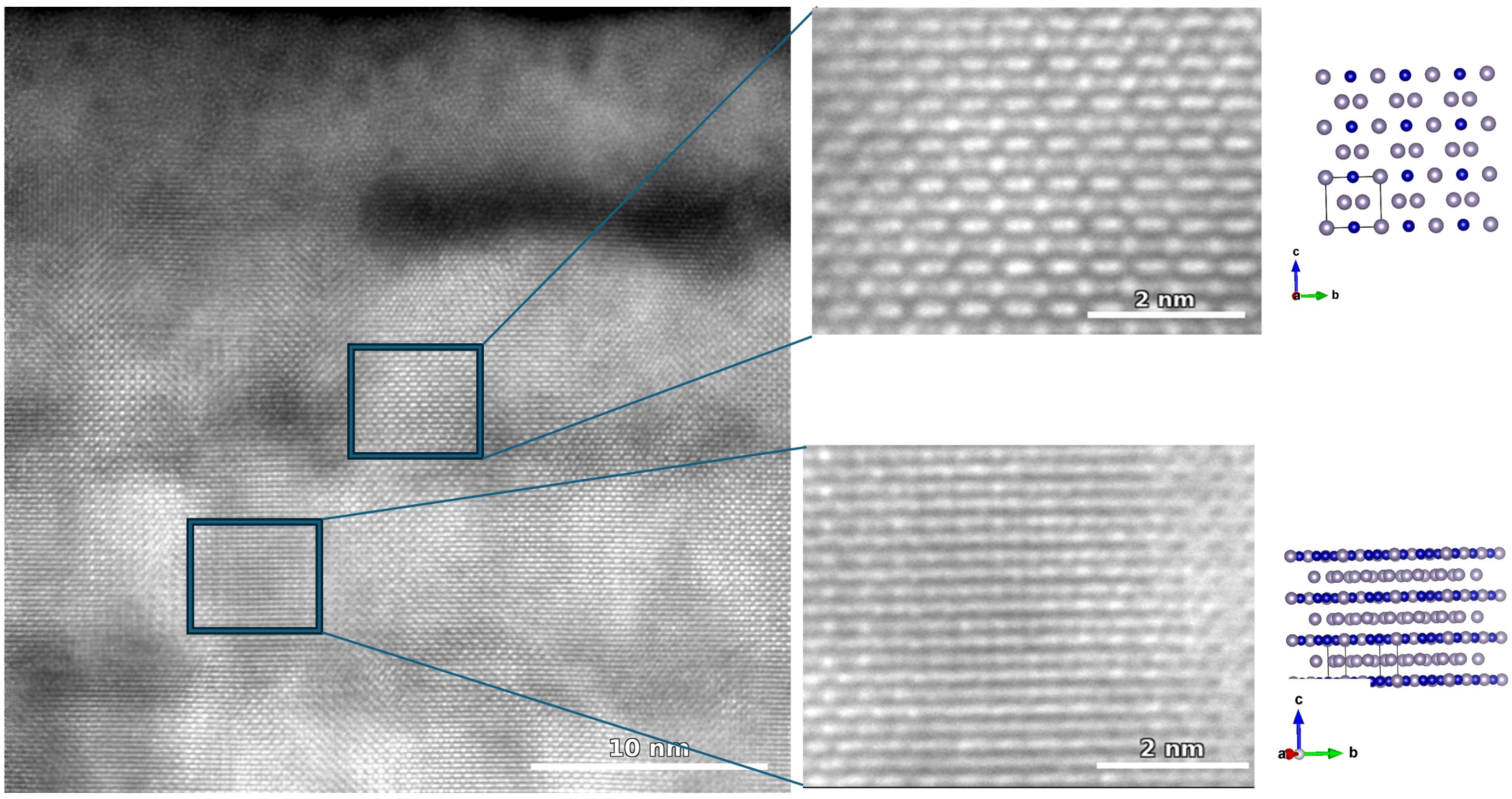

FIG. S9. An example HAADF-STEM image showing structural mosaicity within SL#1.

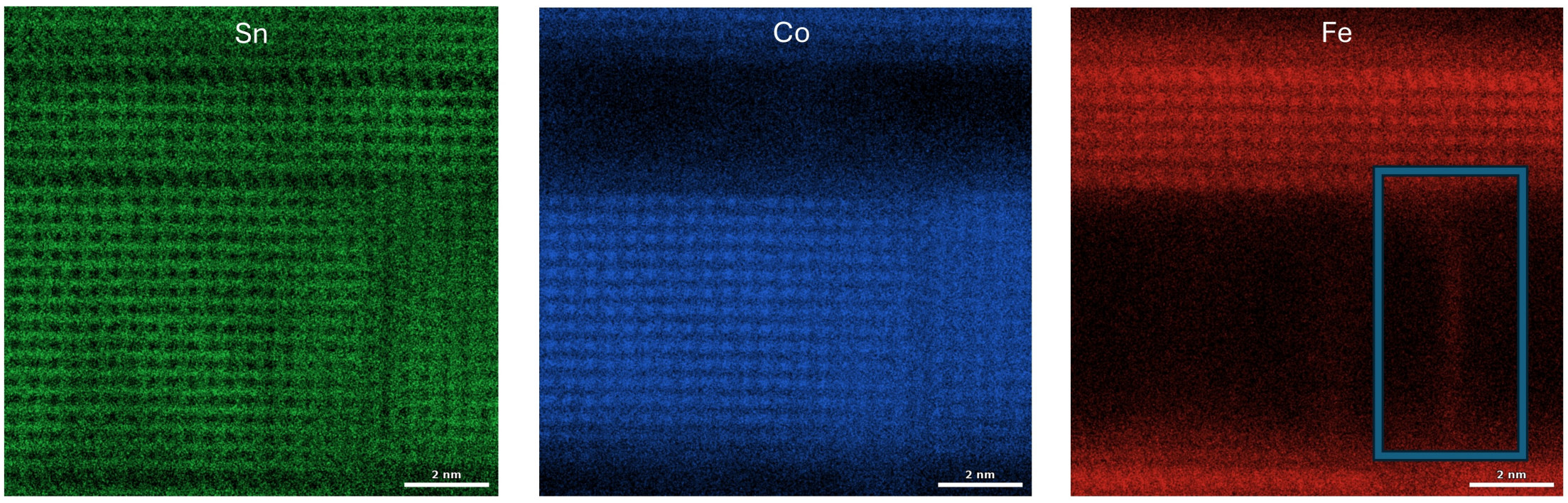

FIG. S10. A local EDS map from SL#1 showing a thin vertical Fe-rich region within CoSn.

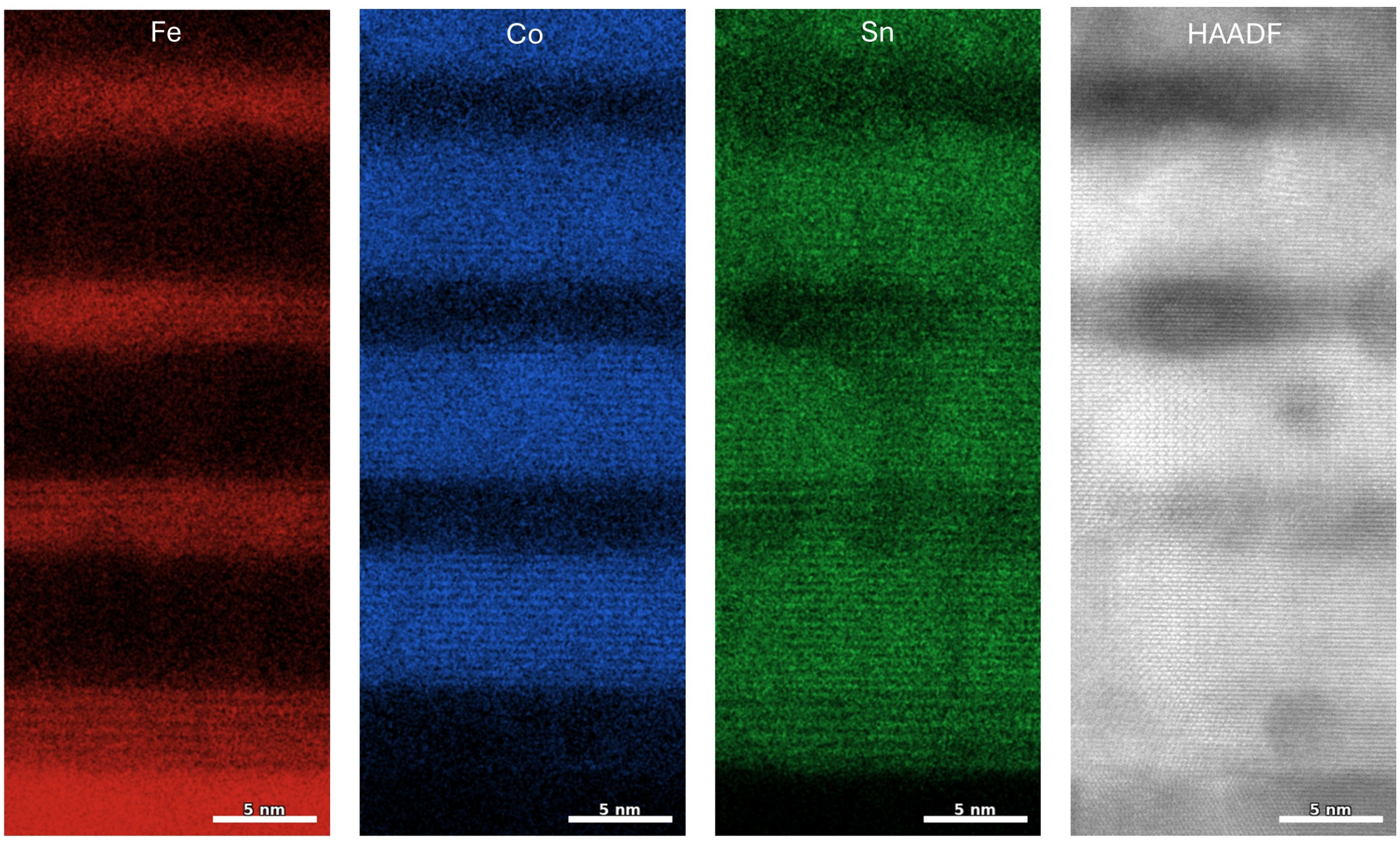

FIG. S11. Example STEM-EDS maps and corresponding HAADF-STEM image in which the darkest top two Fe_3Sn_2 layer have a higher Fe content than the lighter third layer, but all of them contain less Fe than the bottom Fe_3Sn_2 layer that is adjacent to Fe.
